# Constitutionally High Serotonin Tone Favors Obesity: Study on Rat Sublines With Altered Serotonin Homeostasis

**DOI:** 10.3389/fnins.2020.00219

**Published:** 2020-03-25

**Authors:** Maja Kesić, Petra Baković, Marina Horvatiček, Bastien Lucien Jean Proust, Jasminka Štefulj, Lipa Čičin-Šain

**Affiliations:** Laboratory of Neurochemistry and Molecular Neurobiology, Department of Molecular Biology, Ruđer Bošković Institute, Zagreb, Croatia

**Keywords:** serotonin transporter, rat model, obesity, hypothalamus, body weight homeostasis, energy balance

## Abstract

Central and peripheral pools of biogenic monoamine serotonin (5-hydroxytryptamine [5HT]) exert opposite effects on the body weight regulation: increase in brain 5HT activity is expected to decrease body weight, whereas increase in peripheral 5HT activity will increase body weight and adiposity. In a genetic model of rats with constitutionally high- or low-5HT homeostasis (hyperserotonergic/hyposerotonergic rats), we have studied how individual differences in endogenous 5HT tone modulate net energy balance of the organism. The high-5HT and low-5HT sublines of the model were developed by selective breeding toward extreme platelet activities of 5HT transporter, a key molecule determining 5HT bioavailability/activity. In animals from high-5HT and low-5HT sublines, we assessed physiological characteristics associated with body weight homeostasis and expression profile of a large scale of body weight–regulating genes in hypothalamus, a major brain region controlling energy balance. Results showed that under standard chow diet animals from the high-5HT subline, as compared to low-5HT animals, have lifelong increased body weight (by 12%), higher absolute daily food intake (by 9%), and different pattern of fat distribution (larger amount of white adipose tissue and lower amount of brown adipose tissue). A large number of body weight–regulating hypothalamic genes were analyzed for their mRNA expression: 24 genes by reverse transcription–quantitative polymerase chain reaction (*n* = 9–10 rats/subline) including neuropeptides and their receptors, growth factors, transcriptional factors, and receptors for peripheral signals, and a total of 84 genes of various classes by polymerase chain reaction array (pools of six rats/subline). Only few genes showed significant differences in mRNA expression levels between 5HT sublines (e.g. neuropeptide Y receptor, fibroblast growth factor 10), but high-5HT animals displayed a clear trend to upregulation of mRNAs for a number of orexigenic signaling peptides, their receptors, and other molecules with orexigenic activity. Receptors for peripheral signals (leptin, insulin) and molecules in their downstream signaling were not altered, indicating no changes in central insulin/leptin resistance. At the protein level, there were no differences in the content of hypothalamic leptin receptor between 5HT sublines, but significant sex and age effects were observed. Results show that higher constitutive/individual 5HT tone favors higher body weight and adiposity probably due to concurrent upregulation of several hypothalamic orexigenic pathways.

## Introduction

Serotonin (5-hydroxytryptamine [5HT]) in mammalian organism exists in two functionally independent compartments, central and peripheral, which are separated with, for the amine impermeable, blood–brain barrier. In the brain, 5HT is synthesized in neurons of the brainstem raphe nuclei and released into synapse to act as a neuromodulator of many brain circuits. In the periphery, it is produced mostly by intestinal mucosa and released into circulation. Blood platelets transport 5HT throughout and release it on various stimuli to activate 5HT receptors on cells in different peripheral organs mediating thus their functions (Berger et al., 2009). In both compartments, 5HT signaling is ceased by the rapid action of membrane serotonin transporter (5HTT), which (re)uptakes released 5HT back to neurons or into blood platelets. Pharmacological inhibition of 5HTT experimentally or with therapeutic purposes elevates extracellular 5HT level in parallel in both compartments, thus increasing serotonin action in the whole body.

Dysregulation of 5HT signaling is implicated in many pathological conditions, including obesity and other metabolic disorders. The role of the brain 5HT system in the regulation of body weight has been known for a long time (Curzon, 1990), whereas importance of peripheral 5HT in energy homeostasis emerged just recently (Donovan and Tecott, 2013; Namkung et al., 2015; Spohn and Mawe, 2017; Yabut et al., 2019). Soon it became evident that brain and peripheral 5HT systems likely exert opposite effects on metabolic homeostasis (Oh et al., 2016; Wyler et al., 2017).

In the brain, nucleus arcuatus of the hypothalamus is a key structure regulating the body’s metabolic processes. It senses and integrates peripheral signals and adjusts accordingly the expression of orexigenic and anorexigenic peptides. Two major neuronal populations are involved in the appetite regulation: neuropeptide Y (Npy) and agouti-related peptide (Agrp) stimulate feeding and decrease energy expenditure, whereas proopiomelanocortin (Pomc) and cocaine- and amphetamine-related transcript (Cart) have the opposite actions. Both circuits project to downstream hypothalamic nuclei where second-order neuropeptide neurons are located that further control energy homeostasis by releasing regulatory factors such as hypocretin (Hcrt/orexin), melanin-concentrating hormone (Mch), and brain-derived neurotrophic factor (Bdnf) (Timper and Brüning, 2017).

Serotonergic neurons are involved in the control of food intake in a way that increased 5HT signaling suppresses feeding and tend to decrease body weight gain (Curzon, 1990; Lam et al., 2010; Voigt and Fink, 2015). In addition, it increases energy expenditure by enhancing sympathetic outflows to brown adipose tissue (Morrison et al., 2014). Hence, pharmacological elevation of brain 5HT activity has been suggested as a possible antiobesity treatment (Burke and Heisler, 2015). Anorectic 5HT action is exerted through its binding to 5HT1B receptor on hypothalamic Npy/Agrp neurons and to 5HT2C receptors on Pomc/Cart neurons (Heisler et al., 2006). Increased hypothalamic 5HT signaling during development has been suggested to play an important role in a widely acknowledged hypothalamic programming of body mass (Martin-Gronert et al., 2016).

In the periphery, 5HT controls energy homeostasis by acting on various metabolic organs as a gut-derived hormone or autocrine/paracrine signaling molecule (Spohn and Mawe, 2017; Wyler et al., 2017; Yabut et al., 2019). It stimulates adipogenesis (Kinoshita et al., 2010), reduces lipolysis (Sumara et al., 2012), promotes insulin secretion (Paulmann et al., 2009), and inhibits glucose production (Sumara et al., 2012). 5HT also blunts thermogenic activity of brown adipose tissue (Crane et al., 2015; Oh et al., 2015). Pharmacologic inhibition or genetic deletion of a rate-limiting enzyme in peripheral 5HT synthesis protects mice from high-fat diet–induced obesity and insulin resistance (Sumara et al., 2012). Therefore, decreasing peripheral 5HT signaling was suggested as a new strategy for antiobesity treatment (Watanabe et al., 2011; Crane et al., 2015).

The existence of a bimodal control system where central and peripheral increase in 5HT bioavailability exerts the opposite effects on body weight regulation prompted us to investigate how interindividual differences in endogenous 5HT tone will affect net energy balance of the organism. For this purpose, we have used Wistar–Zagreb 5HT (WZ-5HT) rats with constitutionally altered 5HT homeostasis. Two sublines of the model, high-5HT and low-5HT subline, were developed by selective breeding of rats toward naturally occurring extremes of platelet 5HT parameters: platelet 5HT level and maximal velocity of platelet 5HT transporter (Cicin-Sain et al., 1995, 2005; Jernej et al., 1999; Cicin-Sain and Jernej, 2010). Genetic selection of animals for peripheral 5HT resulted also in differences in central 5HT homeostasis between 5HT sublines, as concluded from neurochemical, pharmacological, behavioral, and other functional studies (Romero et al., 1998; Hranilovic et al., 2005; Cicin-Sain and Jernej, 2010; Bordukalo-Niksic et al., 2010). In particular, animals from the high-5HT subline, in comparison to the low 5HT subline, showed higher 5HT turnover in several brain regions, higher hippocampal 5HT recovery *in vivo*, and higher KCl- and citalopram-induced elevation in extracellular 5HT as measured by microdialysis, as well as higher brain ^3^H-citalopram binding as measured by autoradiography. All obtained results indicate different brain/peripheral 5HT system functioning between 5HT sublines. Generally, animals from the high-5HT subline are considered constitutionally hyperserotonergic in comparison to animals the from the low-5HT subline. One of the consequences of described genetic selection was an overweight phenotype of high-5HT animals, associated with bone loss, adiposity, and peripheral insulin resistance (Erjavec et al., 2016). In the present study, we investigated phenotypic/functional differences between animals from 5HT sublines and searched how endogenous, lifelong alteration in 5HT signaling shapes hypothalamic expression status of key body weight–regulating molecules.

## Materials and Methods

### Animals

Studies were performed on two sublines of WZ-5HT rats developed by directed selective breeding toward the extreme values of platelet serotonin level (PSL) and velocity of platelet serotonin uptake (PSU) at the Ruđer Bošković Institute (Zagreb, Croatia). The generation of 5HT sublines has been described previously (Cicin-Sain et al., 1995, 2005; Cicin-Sain and Jernej, 2010). Just in brief, males and females with the highest and the lowest values of platelet 5HT parameters were chosen from a large base population of Wistar rats and mated to generate high-5HT and low-5HT subline, respectively. Determinations of PSL and PSU were performed in offspring of each generation at the age of 5–6 weeks, and animals displaying the extreme values were selected as parents for the next generation. Divergence of mean PSL values stabilized after 5 to 6 generations at approximately 70% (low-5HT subline) and 150% (high-5HT subline) of the mean value of the starting population. In this study, the age of animals varied from 24 h to 9 months (indicated in figures), and both sexes were used. Animals were housed three per cage under controlled conditions (temperature 22°C ± 2°C; humidity 55% ± 10%; 12-h light–dark cycle) with standard rat chow (4RF21, 3.9 kcal/g, 6.55% kcal from fat; Mucedola, Settimo Milanese, Italy) and water *ad libitum*. All experiments were approved by the institutional and national (Ministry of Agriculture, Republic of Croatia) ethical committees and conducted in accordance with the ILAR Guide for the Care and Use of Laboratory Animals and Croatian animal protection law (NN 135/06 and 37/13).

### Measurements of Body Weight, Food, and Water Intake

Body weight of pups was measured 24 h after delivery. Average pup weight was calculated for four litters/subline with comparable number of pups (10–13 pups/litter, 44 pups/subline) and the same ratio of male-to-female offspring, as determined on postnatal day 7.

Milk intake in pups was determined by weight–suckle–weight method (Hernandez et al., 2012). Testing was performed on five occasions (every other day from postnatal days 9–17) in litters with equal number of newborns delivered at the same time of day. At 8 AM, pups were removed from mothers for 4 h. At 12 AM, all pups were weighed, returned to their mothers to nurse for 45 min, and weighed again to estimate milk intake per litter. Milk intake per pup was obtained by dividing total milk intake by the number of pups in each litter.

Food consumption in adults was measured in males aging 5.5 months. At 9 AM, rats were weighted, placed individually in cages, and presented with preweight amount of food. After 24 h, remaining food was weighed, and the amount of food consumed was obtained by subtracting the uneaten food. Daily water consumption was quantified (at 9 AM) using rat volumetric drinking tubes with Lixit valve (Med Associates, Inc., Fairfax, VT, United States). A week prior to experiment, rats were habituated to drinking tubes, and during experiments, they were caged individually. The body length of isoflurane-anesthetized animals was measured from their nose tip to the tail base using millimeter paper.

### Tissue Collection

Animals were sacrificed by decapitation; brains were rapidly removed from the skull and placed with the dorsal side facing the chilled plate for dissection. Using curved forceps and the rat brain stereotaxic atlas (De Groot, 1959) as a guide, a hypothalamus was carefully removed. The curved part of the forceps was pushed down around the hypothalamus, and hypothalamus was gently pinched out (while pushing down with forceps). It was weighted and either immediately frozen in liquid nitrogen for array expression analyses and enzyme-linked immunosorbent assay (ELISA) determination, or placed in RNAlater solution as recommended by the manufacturer (Qiagen, Venlo, Netherlands) for reverse transcription–quantitative polymerase chain reaction (RT-qPCR) analyses. All samples were stored at −80°C until further analyses. Gonadal white adipose tissue (WAT), brown adipose tissue (BAT), liver, heart, and spleen were carefully dissected and weighed.

### Biochemical Measurements

Platelet serotonin level was determined spectrophotometrically, and velocity of PSU was measured radiochemically in the same blood sample obtained from rat jugular vein under isoflurane anesthesia (SomnoSuite anesthesia system; Kent Scientific, Torrington, CT, United States) as described previously (Jernej et al., 1999; Cicin-Sain et al., 2005).

Measurements of selected proteins’ levels were performed by commercial ELISAs. Tissue was homogenized in 1:10 wt/vol tissue protein extraction reagent (T-PER; Thermo Fisher Scientific, Waltham, MA, United States) with protease inhibitor (Halt Protease Inhibitor Cocktail; Thermo Fisher Scientific) added. The resulting lysates were centrifuged (10,000 *g*, 10 min), and the supernatants were assayed for leptin receptor (Lepr), Bdnf, ciliary neurotrophic factor (Cntf), fibroblast growth factor 10 (Fgf10), and 5HTT levels according to manufacturers’ (Elabscience, Wuhan, China) protocols. Assay sensitivities were 18.75 pg/mL for Lepr and Bdnf, 9.38 pg/mL for Fgf10, 7.50 pg/mL for Cntf, and 0.10 ng/mL for 5HTT. Total protein content in homogenates was determined by the Bradford method.

Determination of hypothalamic levels of 5HT and its metabolite 5-hydroxyindoleacetic acid (5HIAA) was performed using high-performance liquid chromatography (HPLC) with electrochemical detection. Tissue samples were homogenized in 10 volumes (vt/wol) of 0.1 M HClO_4_ containing 0.2 mM EDTA and 0.4 mM Na_2_S_2_O_5_ and centrifuged at 14,000 *g* for 15 min at +4°C. Supernatants were filtered, and 20 μL aliquots were injected into the C8 reverse-phase HPLC column (StableBond SB-C8, 3.5 μm, 4.6 × 75 mm; Agilent Technologies, Santa Clara, CA, United States) and protected with guard column (LiChrospher 100 RP-18.5 μm, 4 × 4 mm; Agilent Technologies). The mobile phase contained 0.1 M Na_2_HPO_4_, 0.05 M citric acid, 10% methanol, 0.1 mM EDTA, and 1 mM KCl. The elution was performed at a flow rate of 0.5 mL/min at a pressure of 60 bars. An electrochemical detector (1049A; Hewlett-Packard, Palo Alto, CA, United States) with a glassy carbon electrode was used at +0.60 V versus reference electrode. Quantitative determinations were made by comparison with appropriate external standards using an integrator. The turnover of amine was calculated as the metabolite-to-neurotransmitter ratio.

### Gene Expression Analysis

Total RNA from approximately 30 mg of hypothalamus was isolated with RNeasy Mini Kit (Qiagen) according to manufacturer*’*s protocol including on-column DNA digestion step. Concentration and purity of isolated RNAs were assessed by spectrophotometry (NanoPhotometer; Implen, Westlake Village, CA, United States). A_260_/A_280_ ratio was between 2.16 and 2.34. Aliquots of RNA were run on 1% agarose gel electrophoresis to verify integrity; all samples showed sharp 28S and 18S bands in approximately 2:1 ratio. RNA samples were stored at −80°C until further processing. Relative levels of specific mRNAs were determined using the RT-qPCR based on SYBR Green detection chemistry. cDNA was synthesized from 1,200 ng of each RNA in a final volume of 20 μL using the High-Capacity RNA to cDNA Synthesis Kit (Applied Biosystems, Foster City, CA, United States) according to manufacturer*’*s protocol. Control reactions lacking reverse transcriptase (no-RT) were prepared in order to test genomic DNA contamination. For each tissue, cDNA was synthesized also from the pool of all RNA samples in order to be used for preparing standard curves. cDNAs were diluted to concentration of 6 ng/μL and stored in small aliquots at −20°C. Sequences of the primers used in qPCR are listed in Supplementary Table 1. Quantitative PCR assays were prepared using the Fast SYBR Green Master Mix, in a total volume of 10 μL, and were run on a StepOnePlus Real-Time PCR System (both from Applied Biosystems) according to manufacturer*’*s protocol. Each qPCR plate included a 5-point standard curve with twofold serial dilution, and all reactions were performed in duplicates. Relative expression levels were determined using a relative standard curve method (Larionov et al., 2005) and were normalized to the mean of two reference genes, glyceraldehyde-3-phosphate dehydrogenase (*Gapdh*) and actin beta (*Actb*).

Rat Obesity RT^2^ Profiler PCR Array (Qiagen) was used for measuring expression levels of hypothalamic body weight–regulating molecules in RNA pools obtained from six animals per subline. First-strand cDNA was synthesized from 500 ng of each RNA pool using the RT^2^ First Strand Kit (Qiagen) according to the manufacturer’s instructions. PCR Array assays were prepared using the RT^2^ qPCR SYBR Green/ROX Master Mix (Qiagen) as recommended. Reactions were run on a 7300 Real Time PCR System (Applied Biosystems) employing standard cycling conditions (95°C for 10 min; 40 cycles of 95°C for 15 s and 60°C for 1 min), followed by dissociation curve analysis. Positive PCR control for interassay comparison as well as controls for genomic DNA contamination and reverse transcription all passed quality control. Genes with quantification cycle (C_*q*_) greater than 30 are listed in Table 1 (Complete list of the analyzed genes may be found at^1^). The expression of each gene of interest was normalized to mean of five housekeeping genes (*Rplp1*, *Hprt1*, *Rpl13a*, *Ldha*, and *Actb*). The fold change in relative expression level in high-5HT as compared to low-5HT subline was calculated using the comparative C_*q*_ (ΔΔC_*q*_) method (Livak and Schmittgen, 2001).

**TABLE 1:** Expression level of hypothalamic body weight–regulating molecules in high-5HT and low-5HT sublines of WZ-5HT rats.

Symbol of gene	Description	Quantification cycle		RER (H/L)
		
		High 5HT	Low 5HT	
*Adcyap1*	Adenylate cyclase activating polypeptide 1	23.30	23.37	0.99
*Adcyap1r1*	Adenylate cyclase activating polypeptide 1 receptor 1	23.13	23.64	1.35
*Adipor1*	Adiponectin receptor 1	23.51	23.57	0.98
*Adipor2*	Adiponectin receptor 2	24.66	24.60	0.91
*Adrb1*	Adrenergic, beta- 1-, receptor	28.01	28.63	1.46
*Agrp*	Agouti related protein homolog (mouse)	25.23	25.04	0.83
*Apoa4*	Apolipoprotein A-IV	29.26	28.44	0.54
*Atrn*	Attractin	23.52	23.67	1.05
*Bdnf*	Brain-derived neurotrophic factor	26.03	26.15	1.03
*Brs3*	Bombesin-like receptor 3	28.29	28.91	1.45
*C3*	Complement component 3	27.67	28.39	1.56
*Calca*	Calcitonin-related polypeptide alpha	28.38	29.10	1.57
*Calcr*	Calcitonin receptor	23.63	23.54	0.89
*Cartpt*	CART prepropeptide	21.13	21.11	0.93
*Cck*	Cholecystokinin	24.51	24.92	1.26
*Cckar*	Cholecystokinin A receptor	27.46	27.79	1.18
*Cnr1*	Cannabinoid receptor 1 (brain)	24.16	24.33	1.07
*Cntf*	Ciliary neurotrophic factor	25.82	25.99	1.07
*Cntfr*	Ciliary neurotrophic factor receptor	23.64	24.18	1.38
*Crh*	Corticotropin-releasing hormone	26.15	26.06	0.90
*Crhr1*	Corticotropin-releasing hormone receptor 1	27.04	27.36	1.19
*Drd1a*	Dopamine receptor D1A	27.79	27.57	0.82
*Drd2*	Dopamine receptor D_2_	25.37	25.93	1.39
*Gal*	Galanin prepropeptide	23.54	23.52	0.94
*Galr1*	Galanin receptor 1	25.56	25.35	0.82
*Ghr*	Growth hormone receptor	26.48	26.97	1.33
*Ghrl*	Ghrelin/obestatin prepropeptide	26.09	26.43	1.20
*Ghsr*	Growth hormone secretagogue receptor	26.13	26.02	0.88
*Glp1r*	Glucagon-like peptide 1 receptor	28.25	28.68	1.27
*Grp*	Gastrin-releasing peptide	27.52	27.88	1.22
*Grpr*	Gastrin-releasing peptide receptor	28.36	27.93	0.70
*HcRt*	Hypocretin	22.23	22.70	1.32
*Hcrtr1*	Hypocretin (orexin) receptor 1	27.29	27.78	1.33
*Hrh1*	Histamine receptor H 1	26.93	27.08	1.05
*Htr2c*	5-Hydroxytryptamine (serotonin) receptor 2C	23.99	23.88	0.88
*Il1a*	Interleukin 1 alpha	29.98	29.73	0.80
*Il1r1*	Interleukin 1 receptor, type I	28.14	28.44	1.16
*Il6r*	Interleukin 6 receptor	27.88	28.01	1.04
*Insr*	Insulin receptor	24.94	25.02	1.00
*Lepr*	Leptin receptor	27.56	27.38	0.84
*Mc3r*	Melanocortin 3 receptor	28.09	28.00	0.89
*Mchr1*	Melanin-concentrating hormone receptor 1	26.53	26.59	0.99
*Nmb*	Neuromedin B	26.38	26.67	1.16
*Nmbr*	Neuromedin B receptor	28.73	28.53	0.83
*Nmu*	Neuromedin U	26.52	25.95	0.64
*Npy*	Neuropeptide Y	24.76	24.46	1.07
*Npy1r*	Neuropeptide Y receptor Y1	26.71	26.79	1.00
*Nr3c1*	Nuclear receptor subfamily 3, group C, member 1	24.08	24.22	1.04
*Nts*	Neurotensin	24.37	24.58	1.09
*Ntsr1*	Neurotensin receptor 1	27.25	27.96	1.55
*Oprk1*	Opioid receptor, kappa 1	25.80	25.94	1.05
*Oprm1*	Opioid receptor, mu 1	25.65	25.64	0.94
*Pomc*	Proopiomelanocortin	22.10	22.05	0.92
*Ppara*	Peroxisome proliferator–activated receptor alpha	27.09	27.06	0.92
*Ppargc1a*	Peroxisome proliferator–activated receptor gamma, coactivator 1 alpha	25.19	25.52	1.20
*Prlhr*	Prolactin-releasing hormone receptor	28.45	28.07	0.73
*Ptpn1*	Protein tyrosine phosphatase, non-receptor type 1	25.68	26.04	1.22
*Ramp3*	Receptor (G protein–coupled) activity modifying protein 3	28.32	28.87	1.39
*Sigmar1*	Sigma non-opioid intracellular receptor 1	23.36	23.66	1.17
*Sort1*	Sortilin 1	22.33	22.59	1.14
*Sst*	Somatostatin	21.44	21.58	1.05
*Sstr1*	Somatostatin receptor 1	25.15	25.34	1.08
*Thrb*	Thyroid hormone receptor beta	25.95	25.97	0.96
*Trh*	Thyrotropin-releasing hormone	24.21	24.58	1.22
*Trhr*	Thyrotropin-releasing hormone receptor	26.29	26.28	0.95

*Expression levels were measured in RNA pools obtained from six animals per subline. The expression levels of genes of interest were normalized to mean of five reference genes. Results are shown as relative expression ratio (RER) between high-5HT and low-5HT subline (H/L), and genes are sorted in alphabetical order. C_*q*_ = quantification cycle.*

### Statistical Analysis

Statistical analyses were performed using GraphPad Prism v.0^2^. Normality of data distribution was tested by D’Agostino–Pearson omnibus test, homogeneity of variances by Bartlett test, and presence of outliers by Grubbs test. Means between two groups were compared with two-tailed unpaired Student *t*-test (with Welch correction if variances were significantly different) or Mann–Whitney *U* test, as appropriate. Food intake was analyzed by repeated-measures analysis of variance followed by Fisher least significant difference test. Relationship between biochemical measures was evaluated by Pearson correlation coefficient. Results are presented as individual values and/or group means with standard deviation (SD) or standard error of the mean (SEM). Differences were considered statistically significant if *p* < 0.05.

## Results

**FIGURE 1:**
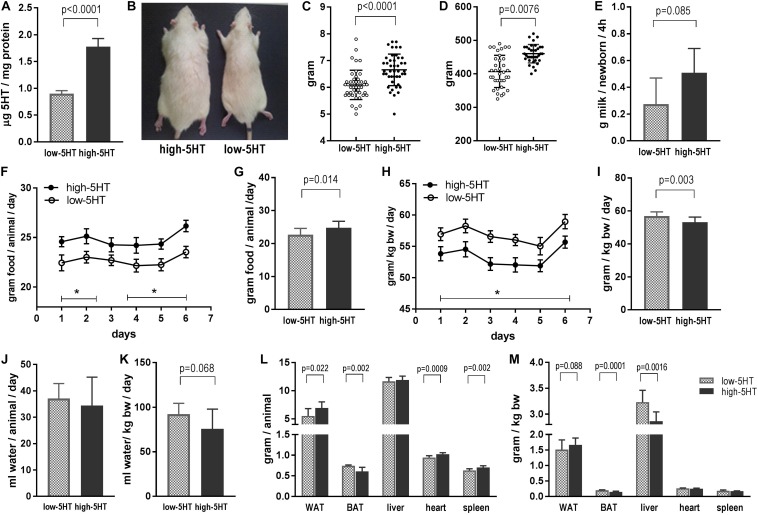
**(A)** Platelet serotonin level in rats from high-5HT and low-5HT sublines. Shown are values for males used in food intake study presented in F–I, but data are representative of all experiments. Mean ± SD, *n* = 9 rats/subline. **(B)** Dorsal view of rats from high-5HT (left) and low-5HT (right) sublines. Males, 9 months of age. **(C)** Body weight of pups from high-5HT and low-5HT litters measured 24 h after delivery. Individual values for pups from four litters per 5HT subline (10–13 pups/litter, equal number of males and females) are presented with indicated mean ± SD. *N* = 44 pups/subline. **(D)** Body weight of male animals from 5HT sublines at the age of 5 months. Individual values, mean ± SD, *n* = 36 rats/subline. **(E)** Milk intake in pups from high-5HT and low-5HT litters (11 pups/litter) measured by weight–suckle–weight method. Average of five measurements from 9 to 17 postnatal days is given ±SD. **(F–I)** Daily food intake in animals from 5HT sublines measured six times in the course of 2 weeks and expressed as total intake (g/animal/day, **F,G**) or relative to body weight of animals (g/kg body weight/day, **H,I**). Mean ± SEM, *n* = 9 rats/subline. Histograms **(G,I)** show averaged six measurements ±SD. **(J)** Daily water intake in animals from 5HT sublines (mL/animal/day) and **(K)** water intake adjusted for their body weight (mL/kg body weight/day). Mean values of four measurements in group of nine animals per subline ± SD are presented. **(L,M)** Weight of gonadal white adipose tissue (WAT), interscapular brown adipose tissue (BAT), liver, heart, and spleen in 4.5-month-old males from low-5HT and high 5HT subline expressed as absolute (g/animal, L) and relative (adjusted for body weight of animal; M) amount. Mean ± SD, *n* = 9 rats/subline.

Phenotypic characteristics of animals from 5HT sublines are shown in Figure 1. Rats from high-5HT and low-5HT subline displayed approximately twofold differences in PSL (high-5HT/low-5HT, H/L = 1.96; Figure 1A) and velocities of PSU (H/L = 1.78). Figure 1B shows dorsal view of a 9-month-old male rat from high-5HT (left) and low-5HT (right) subline.

In comparison to low-5HT rats, high-5HT animals have a higher body weight already at birth (12%, *t* = 4.68, *p* < 0.001; Figure 1C), and similar differences between 5HT sublines were present in adult animals (13%, *t* = 5.75, *p* < 0.001; Figure 1D). There was a highly significant positive correlation between blood 5HT level and body mass of animals from 5HT sublines (*r* = 0.779, *p* < 0.001, *n* = 39; Supplementary Figure 1). Correlation analyses performed separately for high-5HT and low-5HT rats showed significant correlation only in the low-5HT group (*r* = 0.533, *p* = 0.0226, *n* = 19; Supplementary Table 2).

To investigate whether alterations in energy intake are responsible for body weight differences, we measured food intake in pups and in adult animals from 5HT sublines. At the age of 2 weeks, high-5HT pups have tendency to suckle more milk than age-matched low-5HT pups (85%, *t* = 1.959, *p* = 0.085; Figure 1E). In adults, rats from high-5HT subline have significantly higher daily food intake when absolute amount of consumed food is considered (by 9%, *t* = 2.662, *p* = 0.014; Figures 1F,G), whereas their daily food intake adjusted for body weight is significantly lower (by 7%, *t* = 3.291, *p* = 0.003; Figures 1H,I), similarly as their weight-adjusted water intake (by 18%, *t* = 1.958, *p* = 0.068; Figure 1K).

Differences between 5HT sublines in absolute and relative weight of several body organs are shown in Figures 1L,M, respectively. Mass of gonadal WAT was significantly higher (absolute mass by 26%, relative mass by 10%), and mass of interscapular BAT was significantly lower in high-5HT rats (absolute mass by 18%, relative mass by 27%) as compared to low-5HT animals, showing different patterns of fat distribution between 5HT sublines. Visceral and retroperitoneal WAT depots, measured in the same way, were also increased in the high-5HT subline (not shown). Animals from the high-5HT subline had higher weight of heart and spleen (by 8% and 12%, respectively; Figure 1L), as well as longer total body length (by 5%; Supplementary Figure 2).

**FIGURE 2:**
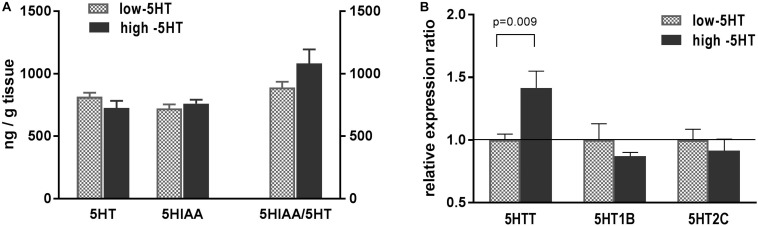
**(A)** Serotonin (5HT), its metabolite (5-hydroxyindoleacetic acid, 5HIAA), and their ratio (5HIAA/5HT) measured in whole hypothalamus of animals from low-5HT and high-5HT sublines. Quantification was performed by HPLC-electrochemical method. Values are mean ± SEM, *n* = 6–7 rats/subline. **(B)** mRNA expression levels of genes for serotonin transporter (5HTT) and serotonin receptors 5HT1B and 5HT2C, measured in hypothalamus of animals from 5HT sublines by RT-qPCR analysis. Expression levels were normalized to mean of two reference genes (*Actb* and *Gapdh*). Results are shown as relative expression ratio between animals from high-5HT and low-5HT subline. Mean ± SEM, *n* = 9–10 rats/subline.

Serotonergic parameters measured in hypothalami of 5HT sublines are shown in Figure 2. Content of hypothalamic 5HT and its metabolite 5HIAA between 5HT sublines did not differ, and there was only a trend (22%, not significant) toward elevation of 5HT turnover in high-5HT rats as compared to low-5HT animals (Figure 2A). Expression analysis of hypothalamic 5HT-related genes revealed significantly increased levels of mRNA for 5HTT (H/L = 1.41, *t* = 2.915, *p* = 0.009) in high-5HT animals, whereas no significant differences between 5HT sublines were present in the expression levels of genes coding for 5HT receptors type 1B (H/L = 0.92) and 2C (H/L = 0.91) (Figure 2B). There was a significant negative correlation between hypothalamic expressions of these two 5HT receptor subtypes (*r* = -0.5295, *p* = 0.0197, *n* = 19; Supplementary Figure 3), which was more pronounced in low-5HT subline (Supplementary Table 3).

**FIGURE 3:**
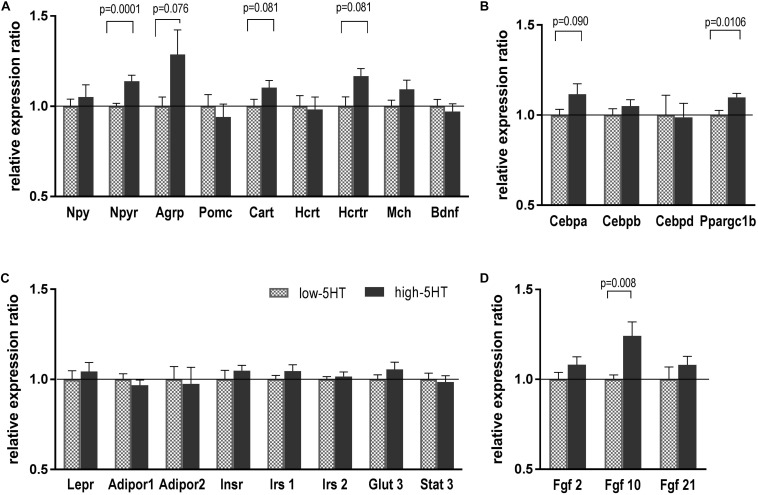
Hypothalamic expression levels of weight-related molecules, neuropeptides, and their receptors **(A)**, transcription factors **(B)**, various peripheral signaling molecules **(C)**, and growth factors **(D)** in animals from 5HT sublines, measured by RT-qPCR analysis. Expression levels were normalized to mean of two reference genes (*Actb* and *Gapdh*) and are shown as relative expression ratio between high-5HT and low-5HT subline. Mean ± SEM, *n* = 9–10 rats/subline. Abbreviations: *Agrp*, agouti-related peptide; *Adipor*, adiponectin receptor; *Bdnf*, brain-derived neurotrophic factor; *Cart*, cocaine-and amphetamine-related transcript; *Cebpa*, *Cebpb Cebpd*, CCAAT/enhancer binding protein alfa, beta, delta; *Fgf*, fibroblast growth factor; *Glut3*, glucose transporter 3; *Hcrt*, hypocretin; *Hcrtr*, hypocretin receptor; *Insr*, insulin receptor; *Irs*, *i*nsulin receptor substrate; *Lepr*, leptin receptor; *Mch*, melanin-concentrating hormone; *Npy*, neuropeptide Y; *Npyr*, neuropeptide Y receptor; *Pomc*, pro-opiomelanocortin; *Ppargc1b*, peroxisome proliferator-activated receptor gamma coactivator 1 beta; *Stat3*, signal transducer and activator of transcription 3.

To investigate which molecules/pathways underlie observed differences between 5HT sublines with regard to body weight and fat distribution, we compared expression levels of various body weight–regulating genes in their hypothalami. As can be seen in Figure 3, no significant differences in mRNA expression levels of hypothalamic neuropeptides were observed between 5HT sublines. There was a trend toward upregulation of *Agrp* and *Cart* mRNAs in high-5HT rats (H/L = 1.29, *t* = 1.887, *p* = 0.076, and H/L = 1.10, *t* = 1.856, *p* = 0.081, respectively). *Npy* receptor mRNA level was significantly higher in high-5HT rats as compared to low-5HT animals (H/L = 1.15, *t* = 3.737, *p* = 0.001) (Figure 3A).

As to hypothalamic expression of transcriptional factors involved in energy expenditure, in high-5HT animals *Ppargc1b* mRNA was significantly increased (H/L = 1.10, *t* = 2.908, *p* = 0.011), and *Cebpa* mRNA showed a tendency toward increase (H/L = 1.12, *t* = 1.755, *p* = 0.090) in comparison to low-5HT animals (Figure 3B).

There were no differences in mRNA expression of receptors/transporters for peripheral signals: *Lepr*, insulin receptor (*Insr*), adiponectin receptors (*Adipor*), glucose transporter 3 (*Glut3*), and in mRNA expression of molecules in their downstream signaling (insulin receptor substrates 1 and 2, signal transducer and activator of transcription 3) between high-5HT and low-5HT animals (Figure 3D). On the other hand, hypothalamic expression of Fgf seems to be upregulated in high-5HT animals (*Fgf10*: H/L = 1.24, *t* = 2.970, *p* = 0.008; *Fgf2* and *Fgf21*: H/L = 1.08, ns) as compared to low-5HT animals (Figure 3D). It should be noted that *p*-values were not corrected for multiple testing, so it should be taken into account that some of the results might be false positives due to type I error.

**FIGURE 4:**
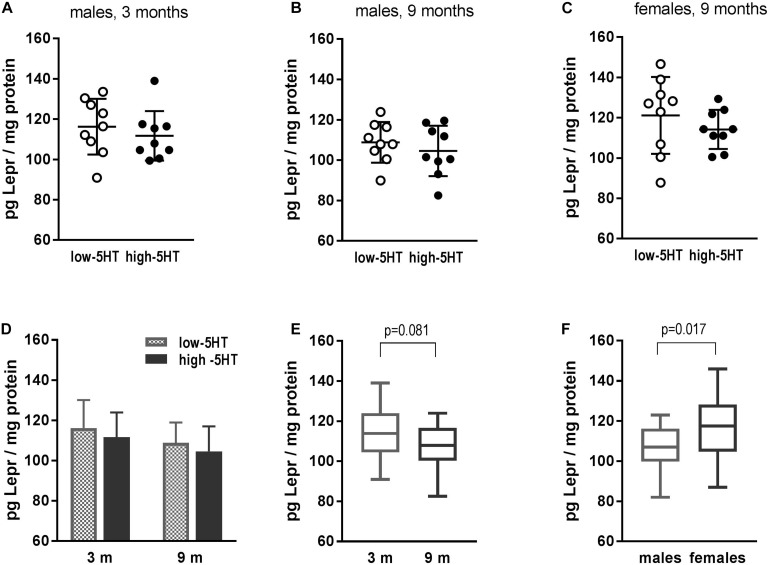
Leptin receptor (Lepr) in hypothalamus of animals from low-5HT and high-5HT sublines, measured by ELISA. **(A,B)** Lepr levels in male rats aged 3 and 9 months. **(C)** Lepr levels in female rats aged 9 months. Data in panel **(A–C)** are presented as individual values with indicated mean ± SD, *n* = 9 rats/subline. **(D)** Comparison of Lepr levels between 5HT sublines in different age groups (same data as **A,B**). There were no differences between age groups in either 5HT subline. Histograms show mean ± SD, *n* = 9 rats/subline. **(E)** Lepr levels in relation to age: males of the same age from both sublines were pooled together, *n* = 18 rats per group. **(F)** Lepr levels in relation to gender: 9-month-old animals of the same sex from both sublines were pooled together, *n* = 18 rats per group. Data in E and F are presented as box-and-whiskers (min–max) plot.

No differences in hypothalamic Lepr between 5HT sublines were shown at the protein level (Figure 4). In all three groups of animals that were analyzed (3- and 9-month-old males and 9-month-old females), practically the same H/L ratio was obtained ranging from 0.94 to 0.96 (Figures 4A–C). There was a tendency of hypothalamic Lepr protein to decrease with age (by 7%, *t* = 1.800, *p* = 0.081; Figure 4E). In addition, gender differences were shown, with females having significantly higher Lepr protein content than males, notwithstanding the 5HT subline (10%, *t* = 2.491, *p* = 0.017; Figure 4F).

**FIGURE 5:**
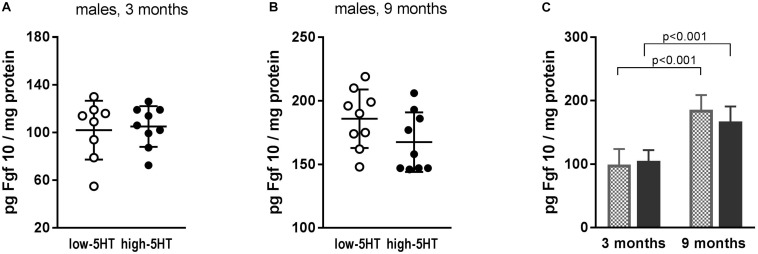
Fibroblast growth factor 10 (Fgf10) levels in hypothalamus of animals from low-5HT and high-5HT sublines, aged 3 months **(A)** and 9 months **(B)**, measured by ELISA. Data are presented as individual values with indicated mean ± SD, *n* = 8–9 rats/subline. Significant age effect **(C)** was shown in both 5HT sublines.

Observed differences between 5HT sublines in *Fgf10* mRNA expression were not confirmed at the protein level (Figure 5). In both 5HT sublines, a significant influence of age was shown, with values being almost twofold higher in older animals (Figure 5B). Hypothalamic protein levels of two more growth factors, Bdnf and Cntf, were also measured, but no differences between 5HT sublines were shown either (not presented).

Limited molecular/biochemical differences observed between 5HT sublines motivated us to compare their hypothalamic gene expression profile in a larger scale, using PCR Array technology. Results of Rat Obesity RT^2^ Profiler PCR Array for genes with quantification cycles higher than 30 are shown in Table 1. Differences in hypothalamic expression levels of body weight–regulating genes between 5HT sublines were in general small, with H/L ratio ranging between 0.54 (apolipoprotein A) and 1.57 (calcitonin-related polypeptide alfa). These data were obtained with RNA pools of six animals per subline and are considered preliminary. Still, there are several interesting observations that will be discussed later that will direct our further studies.

## Discussion

We report that, in our rat model, higher endogenous 5HT tone is associated to increased body weight and adiposity of the animal. The study investigates functional differences and molecular/biochemical changes between constitutively obese high-5HT rats and their constitutively lean counterparts, low-5HT animals. Molecular perturbations in rodent obesity have been studied mostly in relation to experimentally increased caloric intake, while here we studied animals on normal chow diet investigating the impact of endogenous 5HT tone on body weight regulation.

Differences in body weight between 5HT sublines are present already at birth, with low-5HT rats being lighter and never reached up body weight of high-5HT animals (Figures 1C,D). Adult high-5HT animals have a larger amount of gonadal (Figure 1L) as well as visceral and retroperitoneal (not shown) fat and also increased weight of some other organs (e.g. heart, spleen; Figure 1L) in comparison to low-5HT animals. They also have longer femurs (Erjavec et al., 2016) and increased total body length (Supplementary Figure 2). It could be concluded that differences in various tissues/organs concurrently contribute to total body weight differences between 5HT sublines. It is known that 5HT has an important developmental role as a trophic/growth factor (Gaspar et al., 2003) and that maternal 5HT activity impacts fetal development (Côté et al., 2007; Muller et al., 2017). Hence, increased birth weight of high-5HT pups may indicate that maternal high 5HT tone stimulated their *in utero* growth. Alternatively, decreased weight of low-5HT pups may be a consequence of their own 5HT phenotype, similarly to transgenic 5HTT-overexpressing mice with reduced extracellular 5HT, who are smaller and lighter than their wild-type littermates (Pringle et al., 2008). Literature findings on the long-term effects of pharmacologically induced rise in 5HT availability during development on the body weight of animals are rather inconsistent, showing either increased, decreased, or normal body weight in later life (Grzeskowiak et al., 2012; Galindo et al., 2015; De Long et al., 2015). Increased susceptibility to adult obesity was related, among other possible factors, to dysregulation of hypothalamic 5HT system during development (Martin-Gronert et al., 2016).

In hypothalamus of high-5HT rats as compared to that of low-5HT animals, we have shown increased 5HTT mRNA levels (Figure 2), which, based on our previous biochemical/functional findings (Romero et al., 1998; Cicin-Sain and Jernej, 2010), we interpret as an enhanced 5HT synaptic activity (not as an increased 5HT clearance from extracellular space). Relative increase in 5HT turnover in high-5HT rats (H/L = 1.22; Figure 2), although not significant here, is in line with previous results showing elevated metabolic ratio in several brain regions of high-5HT rats and may support their increased brain 5HT activity in comparison to low-5HT animals.

Understanding how endogenous brain 5HTT activity contributes to the body weight regulation is not clear. Similarly to obese high-5HT rats, rodents with genetic deletion of 5HTT have elevated extracellular 5HT levels and tissue 5HIAA/5HT ratios, but findings regarding their obese phenotype are inconsistent (Kim et al., 2005; Homberg et al., 2007; Murphy and Lesch, 2008; Homberg et al., 2010; Zha et al., 2017). Further, human neuroimaging studies support both increase and decrease, as well as no change in intrasynaptic 5HT level in obesity (Koskela et al., 2008; Erritzoe et al., 2010; Hesse et al., 2016; Wu et al., 2017; van Galen et al., 2018). Interestingly, while in morbid obesity no changes in 5HTT binding were observed, moderately obese subjects showed increased 5HTT-binding potential in comparison to lean controls (Koskela et al., 2008). The findings obtained in moderately obese individuals, being in line with results obtained in our high-5HT rats, suggest that they might be a good model for moderate, but not morbid, obesity. Further, in a large group of healthy subjects with varying degrees of adiposity, brain 5HT turnover was chronically elevated in proportion to obesity (Lambert et al., 1999). Recently, an opposite correlation between brain 5HTT bioavailability and body mass index in obese and non-obese subjects was reported (Nam et al., 2018). The complex relationship between 5HT and obesity is further complicated by suggestion of inverse parabolic relationship between 5HTT availability and 5HT level (van Galen et al., 2018). Having in mind reports on the opposite effects of central and peripheral 5HT pools on net energy balance (Namkung et al., 2015; Oh et al., 2016; Wyler et al., 2017; Yabut et al., 2019), our results indicate that in high-5HT animals with endogenously increased endogenous 5HT tone obesogenic mechanisms of peripheral 5HT prevail over anorexigenic action of central 5HT.

As to relation between brain 5HT activity and feeding, pharmacological elevation of synaptic 5HT is repeatedly shown to reduce food intake (Voigt and Fink, 2015), whereas genetic manipulations bring about contrasting results. Thus, both 5HTT knocking-out and 5HTT overexpression result in normal food consumption together with increased and decreased body weight, respectively, Murphy and Lesch (2008), Pringle et al. (2008). In our sublines, the amount of food eaten by the animals correlated with their body weights, so high-5HT rats consume significantly higher absolute amount of food, whereas when adjusted for body weight food intake of high-5HT animals is lower than in their low-5HT counterparts (Figures 1F–I). This suggests important differences in metabolic efficiency between high-5HT and low-5HT animals. Adjusted water intake between 5HT sublines seems to show similar relation as their food intake (Figure 1K). It is known that in rodents food and water intakes are tightly coordinated (Bachmanov et al., 2002), but how interactions between eating and drinking are controlled by the brain is not clear (Zimmerman et al., 2016). Observations on our 5HT sublines indicate that regulation of appetite and thirst may be under mutual coordinated control mediated by 5HT activity.

Observed differences in body weight and food/water intake between 5HT sublines point to dysregulation of hypothalamic mechanisms controlling these functions. Therefore, we have analyzed the expression levels of hypothalamic neuropeptides and their receptors in animals from 5HT sublines. Despite described functional differences, there were no substantial molecular changes in mRNA expression levels of body weight–related hypothalamic peptides. Differences between 5HT sublines, when existed, were small in amount and not quite significant, but by summing up the overall effect, it seems that in high-5HT animals orexigenic signaling peptides are, or tended to be, upregulated in comparison to low-5HT animals (H/L: *Npy* = 1.05; *Npyr* = 1.14, *Agrp* = 1.29; *Hcrt* = 1.06; *Hcrtr* = 1.17; *Mch* = 1.09; Figure 3A), whereas anorexigenic peptides show tendency in the opposite direction [H/L: *Pomc* = 0.94: *Bdnf* = 0.97 (Figure 3) and also *ApoA4* = 0.54; *Crh* = 0.90 (Table 1)]. *Cart* (H/L = 1.10) was suggested to be either orexigenic or anorexigenic, based on the neural circuits that are stimulated (Abdalla, 2017). It is possible that small changes in neuropeptide mRNA expression levels will be more pronounced if specific hypothalamic nuclei were analyzed.

Coexistent increase in mRNA levels of several orexigenic peptides in high-5HT rats suggests an overall increase in the activity of orexigenic pathways and may underlay their susceptibility to develop obesity. The upregulation of Npy protein/mRNA level and/or its release was shown in obese rodents (Kalra et al., 1999; Schaffhauser et al., 2002), as well as in states associated with hyperphagia, such as fasting or lactation (Kalra et al., 1999; Crowley et al., 2004). Increased *Npy* receptor mRNA in high-5HT rats may indicate their increased sensitivity to the effects of Npy, which is the most potent natural orexigenic signal. Similarly, rats prone to diet-induced obesity when fed normal chow have increased hypothalamic *Npy* mRNA expression, as compared with diet-resistant rats (Levin and Dunn-Meynell, 1997). Study of susceptibility of 5HT sublines to develop obesity when fed high-fat diet is in course in our laboratory, and preliminary results show their different functional and molecular response to such metabolic challenge (Kesic et al., 2019).

Alterations in Npy (and other orexigenic peptides) may also contribute to lower basal thermogenic capacity of high-5HT rats because activation of Npy/Agrp neurons was shown to decrease BAT metabolic activity (Morrison et al., 2014; Waterson and Horvath, 2015). Indeed, we have shown earlier that 5HT-high rats display lower interscapular BAT temperature and lower expression of uncoupling protein-1 (Kesic et al., 2018). Here, smaller BAT mass (in both absolute terms and relative to body weight) and altered pattern of fat distribution (increased WAT and decreased BAT mass) in high-5HT animals were demonstrated (Figures 1L,M).

Brain Npy-ergic circuits are probably best-characterized pathways controlling energy homeostasis, but there are also many other hypothalamic neurotransmitter/neuropeptide circuits that complementarily regulate energy balance. By PCR array experiments, we gained broad insight into pathways that may be affected by constitutive alterations in 5HT homeostasis. Array results need to be confirmed by individual PCR experiments, but there are some observations that we would like to note here. Thus, receptors for several anorectic peptides that were suggested, or clinically tested, as an antiobesity treatment (Cron et al., 2016; González et al., 2015; Vu et al., 2015; Li et al., 2016) seem to be upregulated in high-5HT animals (e.g. bombesin-like receptor 3: H/L = 1.45; neurotensin receptor 1: H/L = 1.55; Cntf receptor: H/L = 1.38; adenylate cyclase activating polypeptide 1 receptor type 1: H/L = 1.35; Table 1). This may suggest that individuals with higher 5HT tone would be more sensitive to (therapeutic) administration of corresponding neuropeptides/receptor agonists.

Further, PCR array results indicate that dysregulation of other neurotransmitters involved in hypothalamic control of feeding behavior and energy expenditure, specifically noradrenaline and dopamine pathways, might contribute to obese phenotype of high-5HT rats. Noradrenaline exerts strong orexigenic action through the activation of excitatory β-adrenergic receptors (Adrb) on Npy/Agrp neurons (Paeger et al., 2017), and Adrb1 seems to be upregulated in high-5HT (H/L = 1.46).

Hypothalamic activity depends on various peripheral signals of energy balance among which leptin and insulin are the most important. Both hormones act on arcuate nucleus via inhibition of Npy/Agrp and activation of Pomc/Cart neurons, resulting in reduced food intake and increased energy expenditure (Timper and Brüning, 2017). In contrast to our expectation, there were no differences between 5HT sublines in hypothalamic mRNA expression of either Lepr or Insr or molecules in their downstream signaling pathways (Figure 3C). It is not uncommon that mRNA expression levels do not strictly correspond to the protein level of the respective molecules (e.g. Kappeler et al., 2004). Therefore, we analyzed Lepr protein levels in hypothalami of 5HT sublines, but no differences were observed either (Figure 4). To note, notwithstanding the 5HT subline, significant sex and age differences were demonstrated in Lepr protein levels in hypothalamus, with values being significantly higher in younger than in older animals and in females than in males (Figures 4E,F). Observed sex differences in Lepr protein accord with higher sensitivity of females to the inhibitory effect of leptin on food intake (Clegg et al., 2003) and highlight the importance of evaluation of the effect of potential antiobesity agents on body weight in both sexes. Absence of differences in endogenous insulin/leptin signaling-related mRNA levels in hypothalamus indicates that different adipose phenotype of 5HT sublines is not associated with changes in central insulin/leptin resistance. On the other hand, dysregulation in peripheral insulin signaling was previously reported (Erjavec et al., 2016), with high-5HT animals showing glucose intolerance and insulin resistance in comparison to low-5HT subline.

Among molecules recently recognized as regulators of metabolic functions, adipogenesis, and energy homeostasis, several members of Fgfs (Fgf10, Fgf19, Fgf21) were also suggested as candidates for novel antiobesity drugs (Ohta and Itoh, 2014; Kyrou et al., 2017). In search for body weight–regulating mechanisms affected by constitutional differences in 5HT tone, we analyzed mRNA expression levels of several Fgfs in hypothalami of 5HT sublines and showed their upregulation in high-5HT subline. However, significant increase in Fgf10 mRNA expression (H/L = 1.24; *p* = 0.008; Figure 3D) was not paralleled with protein changes (Figure 5), indicating a tight control of its level. The effect of aging on hypothalamic Fgf10 protein level (Figure 5) is in line with its implication in adult brain neurogenesis and, to our knowledge, is reported for the first time.

## Conclusion

In conclusion, by using selectively bred WZ-5HT rat sublines, we have shown that constitutive 5HT tone is one of the endogenous factors contributing to, or protecting from, becoming obese. In particular, our results show that low endogenous 5HT tone supports the maintenance of low body weight and low adiposity. The important advantage of our rat model is that the difference in platelet 5HTT activity between 5HT sublines is approximately twofold, similarly to the degree of variation in the level of 5HTT expression in healthy human population (Malison et al., 1998; Murphy and Lesch, 2008). Hence, our model probably mimics well-human physiological situation.

## Data Availability Statement

All datasets generated for this study are included in the article/Supplementary Material.

## Ethics Statement

The animal study was reviewed and approved by the Bioethic Committee of the Ruđer Bošković Institute and Bioethic Committee of the Ministry of Agriculture, Republic of Croatia.

## Author Contributions

LČ-Š conceived the project, designed the experiments, and wrote the manuscript. JŠ supervised and analyzed the molecular experiments, and critically revised the first draft of the manuscript. MK, PB, MH, and BP performed and analyzed the experiments. All authors discussed results and have approved the final version of the manuscript.

## Conflict of Interest

The authors declare that the research was conducted in the absence of any commercial or financial relationships that could be construed as a potential conflict of interest.

## Footnotes

1 www.qiagen.com

2 www.graphpad.com
