# Supplementary material for: Constitutionally High Serotonin Tone Favors Obesity: Study on Rat Sublines With Altered Serotonin Homeostasis
Source: Front Neurosci. 2020 Mar 25;14:219. doi: 10.3389/fnins.2020.00219 (PMC7109468; doi:10.3389/fnins.2020.00219)
Supplement: Supplementary file 1 [file Data_Sheet_1.PDF]

SUPPLEMENTARY METHODS

Supplementary Table 1. Primer sequences used in RT-qPCR analysis

NCBI symbol	Gene	Forward primer sequence	Reverse primer sequence
Actb	Actin beta	5’-GCGCAAGTACTCTGTGTGGA	5’-ACATCTGCTGGAAGGTGGAC
Adipor1	Adiponectin receptor 1	5’-GCTGGCCTTTATGCTGCTCG	5’-TCTAGGCCGTAACGGAATTC
Adipor2	Adiponectin receptor 2	5’-CCACAACCTTGCTTCATCTA	5’-GATACTGAGGGGTGGCAAAC
Agrp	Agouti related protein	5’-GCAGAGGTGCTAGATCCACAGAA	5’-AGGACTCGTGCAGCCTTACAC
Bdnf	Brain derived neurotrophic factor	5’-GAAAGTCCCGGTATCCAAAAG	5’-CGCCACCCAATTCTCTTTTTG
Cart	Cocaine- and amphetamine-regulated transcript protein	5’-GCCAAGTCCCCATGTGTGAC	5’-CACCCCTTCACAAGCACTTCA
Cebpa	CCAAT enhancer binding protein (C/EBP), alpha	5’-GACCATCCGCCTTGTGTGTA	5’-CTGACATTGCACAAGGCACC
Cebpb	CCAAT/enhancer binding protein (C/EBP), beta	5’-GACAAGCTGAGCGACGAGTA	5’-AGCTGCTCCACCTTCTTCTG
Cebpd	CCAAT/enhancer binding protein (C/EBP), delta	5’-GAATTGCTACAGTTTCTTGG	5’-ATGCGCAGTCTCTTCCTC
Fgf2	Fibroblast growth factor 2	5’-TTCACAGCCTGTGCTCTAGGG	5’-GATCGGGTCAGGTTTTGGAAA
Fgf10	Fibroblast growth factor 10	5’-GAGATGTCCGCTGGAGAAAG	5’-CCCCTTCTTGTTCATGGCTA
Fgf21	Fibroblast growth factor 21	5’-AGGCTTTGACACCCAGGATT	5’-ACAGATGACGACCAGGACAC
Gapdh	Glyceraldehyde-3-phosphate dehydrogenase	5’-TGCCCCCATGTTTGTGATG	5’-TGGTGGTGCAGGATGCATT
Glut3	Glucose transporter 3	5’-TGGCTACAACACCGGAGTCATCAA	5’-CTGCCAAAGCGGTTGACAAAGAGT
Hcrt	Hypocretin neuropeptide precursor	5’-TAGAGCCATATCCCTGCCCT	5’-GCGAGGAGAGGGGAAAGTTAG
Hcrtr	Hypocretin receptor 1	5’-GCGCGATTATCTCTATCCGAA	5’-AAGGCTATGAGAAACACGGCC
Htr1b	5-hydroxytryptamine receptor 1B	5’-CTGCTAAAAGAACTCCCAAAA	5’-TTGGGTGTCTGTTTCAAAATC
Htr2c	5-hydroxytryptamine receptor 2C	5’-GCAAGCATAGGCCAACGAAC	5’-TTAGGTGCATCAGGAGCGAG
Insr	Insulin receptor	5’-ATCTCCTGGGATTCATGCTG	5’-TACTGGGTCCAGGGTTTGAG
Irs1	Insulin receptor substrate 1	5’-GATTTAAGCACCTATGCCAG	5’-GAATCGTGAAAGAGTTCGAG
Irs2	Insulin receptor substrate 2	5’-CCACACACCTGTCCTCATTG	5’-TAATCCGCTTTGCCAAAATC
Lepr	Leptin receptor	5’-AGGCCCAGACATTTTTCCTT	5’-TTCCAAAAGCTCATCCAACC
Mch	Melanin-concentrating hormone	5’-ATCGGTTGTTGCTCCTTCTCTG	5’-TCTGCTTGGAGCCTGTGTTCTT
Npy	Neuropeptide Y	5’-AGAGATCCAGCCCTGAGACA	5’-TCACCACATGGAAGGGTCTT
Npyr	Neuropeptide Y receptor	5’-GCTGTGGAACGTCATCAGCTA	5’-TTGATAGATCACGAAGGGCAG
Pomc	Proopiomelanocortin	5’-GAGGTTAAGGAGCAGTGACTAAGA	5’-GTAGCAGAATCTCGGCATCTTCC
Ppargc1b	Peroxisome proliferative activated receptor, gamma, coactivator 1 beta	5’-CTACCAGAGCCCACCCAGTA	5’-CAGGATGAGGAGCCAGAACT
Slc6a4	Solute carrier family 6 member 4	5’-TCTGAAAAGCCCCACTGGACT	5’-TAGGACCGTGTCTTCATCAGGC
Stat3	Signal transducer and activator of transcription 3	5’-TCGGAAAGTATTGTCGCCCC	5’-GACATCGGCAGGTCAATGGT

SUPPLEMENTARY RESULTS

Supplementary Figure 1.

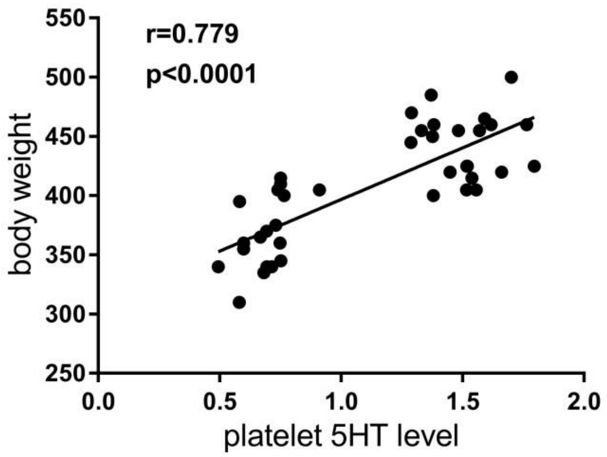

Correlation analysis between platelet 5HT level (µg 5HT/mg platelet protein) and body weight (grams) of animals from high-5HT and low-5HT sublines.
r= Pearson correlation coefficient

Supplementary Table 2. Correlation analyses between blood 5HT level and body mass of animals. Data from Supplementary Figure 1.

Rat subline	No of animals	Correlation coefficient	p-value
All animals	39	0,779	0,0001
High-5HT subline	20	-0,037	0,8731
Low-5HT subline	19	0,533	0,0226

Supplementary Figure 2.

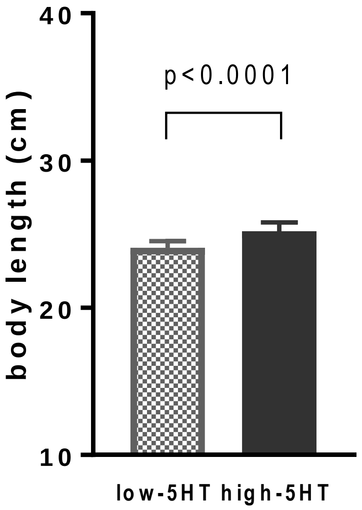

Body length of male animals from 5HT-sublines at 9 month of age as measured from nose tip to tail base. N=14-16 rats/subline, M±SD.

Animals from high-5HT subline have significantly longer body than low-5HT animals (H/L = 1.046, t=5.805, $p<0.0001$).

Supplementary Figure 3.

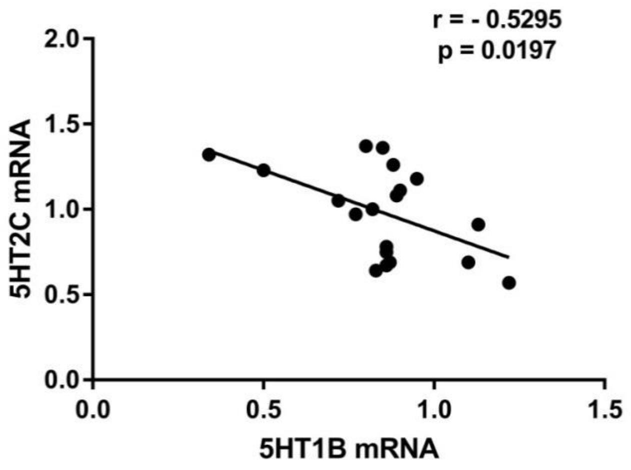

Correlation analysis between hypothalamic expression of 5HT1B and 5HT2C expression of animals from high-5HT and low-5HT sublines
N=10 rats/subline, r= Pearson correlation coefficient

Supplementary Table 3. Correlation analyses between hypothalamic expression of 5HT1B and 5HT2C receptors. Data from the Supplementary Figure 3.

Rat subline	No of animals	Correlation coefficient	p-value
All animals	20	-0,5295	0,0197
High-5HT subline	10	-0,5686	0,0863
Low-5HT subline	10	0,6923	0,0388
